# Evaluating the Clinical Value of Oblique-View Radiographs in the Initial Intervention of Closed Distal Radius Fractures: An External Survey of Hand Surgeons

**DOI:** 10.1016/j.jhsg.2022.09.005

**Published:** 2022-10-28

**Authors:** Michael Orcutt, Todd Foster, Thomas Fischer

**Affiliations:** ∗ Clinical Research Chair, Marian University College of Osteopathic Medicine, Indianapolis, IN; † Department of Clinical Research, Ascension St. Vincent, Indianapolis, IN; ‡ Indiana Hand to Shoulder Center, Indianapolis, IN

**Keywords:** Hand surgery, Musculoskeletal radiograph, Orthopedic trauma, Treatment algorithms, Wrist trauma, wrist fracture

## Abstract

**Purpose:**

Traditionally, an acute wrist radiograph series comprises posteroanterior, oblique, and lateral projections. There is controversy within the field of orthopedics, however, over the value of the oblique view in determining a plan of care for a given fracture. An external survey of practicing hand surgeons was conducted to evaluate whether the addition of the oblique view to a 2-view (posteroanterior and lateral) series resulted in a difference in the initial intervention plan for closed distal radius fractures (DRFs).

**Methods:**

Participants, licensed and practicing hand surgeons in the United States, reviewed 30 sets of wrist radiograph studies twice (once as a complete 3-view series and again with the oblique omitted) in a randomized order. Cases were randomly selected to include 10 pediatric, 10 geriatric, and 10 intermediate/adult DRFs. After reviewing the films and demographic information, the participants selected their preferred initial intervention from the following list: (1) treatment using a cast or orthosis without reduction, (2) closed reduction under or without fluoroscopy with treatment using a cast or orthosis, (3) closed reduction and percutaneous fixation with treatment using a cast or orthosis, and (4) open reduction internal fixation and subsequent treatment in a cast or orthosis.

**Results:**

A calculated Cohen kappa for the entire sample revealed a statistically significant and strong association between 2- and 3-view survey answers (κ = 0.81, *P* < .001*).* Each pairing was examined separately, and 90% of the questions had a statistically significant (*P* < .05) Cohen kappa pairing; however, many were in the 0.5–0.75 range.

**Conclusions:**

The data support the hypothesis that the addition of an oblique view radiograph to a posteroanterior and lateral series does not change surgical decision-making in the initial evaluation of acute closed DRF in many situations. However, although most cases had a statistically significant (*P* < .05) agreement between the surveys, the oblique view did result in some meaningful intervention changes. Therefore, the elimination of the oblique view cannot be supported by our findings.

**Clinical relevance:**

This is a decision analysis survey study designed to investigate how the oblique-view radiograph influences DRF surgical decision-making.

Distal radius fractures (DRFs) rank among the most common fractures in American populations, accounting for approximately 25% of pediatric fractures and 18% of geriatric fractures.^1^ With trending increases in wrist fracture incidence paralleling increased incidence in osteoporosis and an aging patient population, the cost of care for these lesions amounts to a considerable burden to both private payers and the Medicare system. In 2007, Medicare distributed $170 million for the management of DRFs, with some studies estimating that number to double in the next decade if intervention trends continue.^2^, ^3^, ^4^ This does not include management of all suspected fractures and is likely an underestimation of the overall cost that initial management of wrist injuries poses to the health care system.

Radiographic visualization of the wrist is the gold standard for diagnosis in the setting of acute wrist injury with a suspected fracture. Historically, the 3-view wrist series (posteroanterior [PA], lateral, and oblique) is the study of choice in this situation.^5^ Anecdotally, physicians have reported infrequent reviews of the oblique view of this series, instead relying more heavily on the PA and lateral projections to diagnose fractures and develop a treatment plan. Additionally, there is the understanding that additional views could be obtained in the operating or procedure room if it is determined that the patient will be taken for open reduction internal fixation or reduction under fluoroscopy. Although the oblique-view radiograph is by no means the costliest aspect of care for DRFs, x-rays are the initial study of choice for acute presentation following physical examination and mechanism of injury consistent with the risk of wrist fracture. If deemed redundant in this setting, elimination of the oblique view would reduce the cost of evaluation for individuals presenting with the chief complaint of wrist pain requiring radiographs, regardless of whether they are ultimately diagnosed with a fracture.

With all this in mind, an external survey was developed and conducted to evaluate the clinical impact of the oblique-view radiograph in the setting of DRFs. This study was performed with the hypothesis that there would be statistically significant similarities in the initial treatment plans chosen by hand surgeons for the management of closed DRFs when provided either a 3-view wrist series or the same series without the oblique view. In other words, the study predicted that the addition of the oblique view would not change the initial treatment plan chosen for acute closed DRF.

## Material and Methods

The survey consisted of 10 pediatric (patients aged 4–16 years), 10 geriatric (patients aged >65 years), and 10 intermediate (patients aged 17–64 years) wrist x-ray series. The age restrictions were chosen to reflect the bimodal distribution of DRF incidence, with the separation of age groups reflecting the nuances of treatment methodology for pediatric and geriatric cases. Although the intermediate age group represents a relative minority of overall DRFs, it was ultimately included to not exclude any portion of the population. Each question on the survey displayed the age and sex of the patient in addition to wrist films from their initial posttraumatic encounter. The 2 surveys consisted of the same 30 cases; the first survey displayed only PA and lateral view x-rays serving as the experimental survey, and the second survey included complete 3 view x-ray series serving as the control. The participants had to complete the 3-view survey before starting the 2-view survey, and the order in which cases were presented in each survey was randomized within Research Electronic Data Capture. For each question, subjects reviewed included films and demographic information before selecting their initial treatment plan from the following list: treatment in plaster without reduction, the closed reduction under or without fluoroscopy with treatment using a cast or orthosis, closed reduction and percutaneous fixation with treatment using a cast or orthosis, and open reduction internal fixation and subsequent treatment using a cast or orthosis. The cases included in the study were selected via a random data pull of patient encounters from a pool of encounters between January 1, 2018, and December 31, 2019, with the diagnosis of DRF, initial encounter (*International Classification of Diseases, Tenth Revision* codes S52.501A and S51.502A). To be considered for the study, the patients had to meet the aforementioned age inclusion criteria in addition to having been discharged from physician care for the fracture in question at the time of the data pull. Additionally, included initial encounters must have taken place within 3 days of the reported trauma. Table 1 displays the demographic characteristics of the patients included in the survey and the question pairing. Although cases were presented in a randomized order, pairs were tagged to perform the analysis post hoc.

**Table 1:** Question Pairing Characteristics of the Patients Reviewed in the Survey

Patient Image Sex	Patient Image Age, y	2 Radiographs	3 Radiographs	Pairing
F	68	Q1	Q31	Pair 1
M	68	Q2	Q32	Pair 2
F	73	Q3	Q33	Pair 3
F	65	Q4	Q34	Pair 4
F	70	Q5	Q35	Pair 5
F	71	Q6	Q36	Pair 6
F	66	Q7	Q37	Pair 7
F	69	Q8	Q38	Pair 8
F	66	Q9	Q39	Pair 9
M	66	Q10	Q40	Pair 10
M	20	Q11	Q41	Pair 11
F	32	Q12	Q42	Pair 12
M	39	Q13	Q43	Pair 13
F	48	Q14	Q44	Pair 14
M	20	Q15	Q45	Pair 15
F	39	Q16	Q46	Pair 16
M	18	Q17	Q47	Pair 17
F	22	Q18	Q48	Pair 18
M	25	Q19	Q49	Pair 19
M	20	Q20	Q50	Pair 20
M	16	Q21	Q51	Pair 21
F	11	Q22	Q52	Pair 22
F	10	Q23	Q53	Pair 23
F	11	Q24	Q54	Pair 24
F	9	Q25	Q55	Pair 25
M	14	Q26	Q56	Pair 26
M	7	Q27	Q57	Pair 27
M	15	Q28	Q58	Pair 28
M	14	Q29	Q59	Pair 29
M	14	Q30	Q60	Pair 30

The survey was constructed in Research Electronic Data Capture following institutional review board approval at the home institution and was distributed to 110 hand surgeons practicing across 12 states in various subspecialties in April 2021.^6^ The participants included members of a monthly hand trauma case review group in addition to colleagues and current and former partners of the authors. The survey remained open for 3 months for the participants to complete at their convenience. In total, 32 participants completed both components of the survey, which took approximately 45 minutes to complete in its entirety. The participants were not informed of the premise of the study until after the survey had closed to all participants.

It should be noted that subjects were not evaluated regarding which treatment plan they chose but rather whether or not their decisions remained consistent between the control and experimental groups (the 3-view and 2-view radiograph series, respectively). The 2-view survey was always completed before the 3-view survey to reduce potential recall bias resulting from the memory of the oblique view. Cohen kappa was calculated to assess the level of agreement between the treatment options selected across all providers when comparing their responses on the 2- to 3-view. Cohen kappa was calculated here to determine the intrarater reliability of how each provider answered each question. A reliable test taking population, per Cohen kappa, means that individuals answered paired 2- and 3-view questions with the same intervention. As Cohen kappa approaches 1, a population of test takers becomes increasingly reliable, that is, they answer the paired questions the same way. It should be noted that Cohen kappa ranges from −1 (perfect disagreement) to 1 (perfect agreement). Cohen kappa was calculated for all responses among all patients and for each patient reviewed. Pearson chi-square or Fisher exact tests were completed for the age and sex groups to determine whether the distribution in the level of agreement was significantly different. A *P* value of .05 was established as the α threshold for the inferential tests performed in this study. SPSS 24.0 was used to complete the analysis.^7^

## Results

### Participation and demographic characteristics

Of the 110 physicians to whom the survey was distributed, 32 completed it in its entirety (29.1% participation). Of the surgeons who completed the survey, 21 self-identified as subspecialists in hand and/or upper extremity surgery (Table 2). The participants were majority male (n = 31, 96.9%) and European American (n = 23, 71.9%), with a mean participant age of 50.8 years and a mean practice length of 18.3 years (Table 3).

**Table 2:** Participant Self-Identified Subspecialization

Subspecialty	n	%
Hand and/or upper extremity surgery	21	65.6
Other	11	34.4
Total	32	100.0

**Table 3:** Physician Participant Demographic Characteristics

Demographic	95% CI for Mean			
	n	Mean (SD)	Lower	Upper
Age of the physician rater, y	32	50.84 (11.37)	46.74	54.94
Physician rater years of practice	32	18.34 (10.91)	14.41	22.28

	n	%
Men	31	96.9%

Ethnicity		

European American	23	71.9%
African American	2	6.3%
Asian American	3	9.4%
Other	4	12.5%

### Variation between individual participants

Cohen kappa was calculated for all cases collectively in addition to individual analysis of each of the 30 paired cases (Tables 4, 5). This metric is used to quantify the rates of agreement for individual participants while also accounting for random chance. In simplified terms, Cohen kappa treats the 2 surveys as identical and evaluates intraobserver error between the 2 surveys. Cohen kappa also stratifies the degree of agreement found in the included legend of Table 4. Of the 30 pairs, kappa interpretations found 6 pairs in perfect agreement, 2 pairs in almost perfect agreement, 12 pairs in substantial agreement, 6 pairs in moderate agreement, 2 pairs in fair agreement, and 2 pairs in poor agreement (Table 4). Collectively, there were 957 pairs of answers among the 32 responding physicians. The Cohen kappa calculated for the entire sample was “almost perfect” (κ = 0.81, *P* < .001). Table 5 displays a crosstabulation of the entire field of questions. Overall, there was positive agreement in 90% of the paired cases. There were 3 instances where a physician rated a 2-view question but did not answer the matched 3-view question.

**Table 4:** Cohen Kappa for Intraobserver Error Among the Question Pairings∗

Pairing	Cohen Kappa	*P* Value	Interpretation
Overall (N = 957)	0.81	<.001	Almost perfect
Pair 1	0.49	<.001†	Moderate
Pair 2	0.57	<.001†	Moderate
Pair 3	0.03	0.86	Poor
Pair 4	N/A	N/A (constant)‡	Perfect
Pair 5	0.67	<.001†	Substantial
Pair 6	0.65	<.001†	Substantial
Pair 7	0.65	<.001†	Substantial
Pair 8	0.65	<.001†	Substantial
Pair 9	0.75	<.001†	Substantial
Pair 10	0.66	<.001†	Substantial
Pair 11	0.53	<.001†	Moderate
Pair 12	0.25	.02†	Fair
Pair 13	0.31	.05	Fair
Pair 14	N/A	N/A (constant)‡	Perfect
Pair 15	0.63	<.001†	Substantial
Pair 16	0.56	<.001†	Moderate
Pair 17	0.73	<.001†	Substantial
Pair 18	N/A	N/A (constant)‡	Perfect
Pair 19	N/A	N/A (constant)‡	Perfect
Pair 20	0.69	<.001†	Substantial
Pair 21	0.75	<.001†	Substantial
Pair 22	0.89	<.001†	Almost perfect
Pair 23	N/A	N/A (constant)‡	Perfect
Pair 24	0.82	<.001†	Almost perfect
Pair 25	N/A	N/A (constant)‡	Perfect
Pair 26	0.64	<.001†	Substantial
Pair 27	0.65	<.001†	Substantial
Pair 28	−0.05	.74	Poor
Pair 29	0.55	<.001†	Moderate
Pair 30	0.57	<.001†	Moderate

∗ Alternative paired analysis of 2- and 3-view agreement. Note that Cohen kappa cannot be calculated for identical pairs; therefore, N/A signifies perfect agreement. Cohen kappa interpretation: <0.00 indicates poor, 0.00–0.20 indicates slight, 0.20–0.40 indicates fair, 0.41–0.60 indicates moderate, 0.61–0.80 indicates substantial, and 0.81–1.00 indicates almost perfect.

† Statistical power.

‡ N/A signifies perfect agreement. No value is assigned as the calculation involves a zero in the denominator.

**Table 5:** Crosstabulation of the 2- and 3-View Selected Treatment Options

			Collective Responses to Three View Survey Questions				Total
			Treatment in Split/Cast Without Reduction	Closed Reduction Under or Without Fluoroscopy With Treatment in Split/Cast	Closed Reduction and Percutaneous Fixation With Treatment in Split/Cast	Open Reduction Internal Fixation With or Without Subsequent Treatment in Split/Cast	
Collective Responses to Two View Survey Questions	Treatment in orthosis/cast without reduction	n	448	16	6	17	487
		% of total	46.8%	1.7%	0.6%	1.8%	50.9%
	Closed reduction under or without fluoroscopy with treatment in orthosis/cast	n	9	92	12	11	124
		% of total	0.9%	9.6%	1.3%	1.1%	13.0%
	Closed reduction and percutaneous fixation with treatment in orthosis/cast	n	6	2	50	8	66
		% of total	0.6%	0.2%	5.2%	0.8%	6.9%
	Open reduction internal fixation with or without subsequent treatment in orthosis/cast	n	17	5	7	251	280
		% of total	1.8%	0.5%	0.7%	26.2%	29.3%
Total		n	480	115	75	287	957
		% of total	50.2%	12.0%	7.8%	30.0%	100.0%

Each individual paired question was also examined for agreement among the physician raters. Statistically significant positive agreement was found for every question in the series except for 3 questions (pair 3, 13, and 28) (*P* ≥ .05), resulting in a statistically significant agreement in 90% of the questions. Five of the paired questions were found to be constant, meaning there was perfect (100%) agreement in the choice of the treatment plan.

### Consistency of agreement based on sex and age

**Figure 1:**
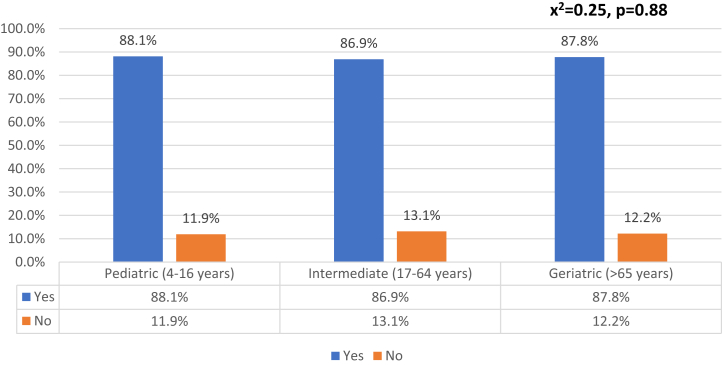
Rates of agreement between interventions selected for 2-view and 3-view radiograph series stratified by patient age demographic. There were found no statistically significant difference in the rates of agreement for each age group (x^2^ = 0.25, *P* = .88).

**Figure 2:**
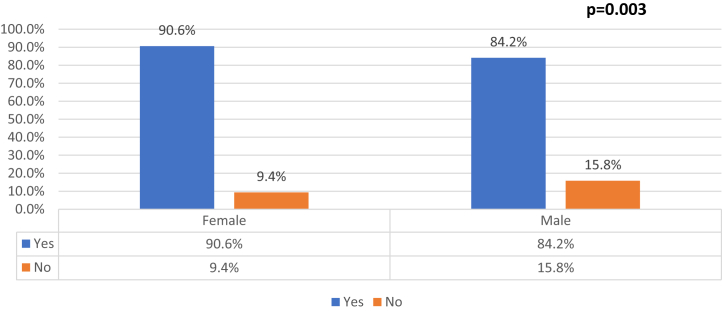
Rates of agreement between interventions selected for 2-view and 3-view radiograph series stratified by patient sex. There were statistically significant rates of agreement for male and female patients (*P* = .003).

Analysis was also performed to investigate whether patient age and sex independently influenced treatment plan agreement for the 2- and 3-view surveys. Pearson chi-square analysis of the rates of agreement for the 3 patient age subcategories found no statistically significant difference in the rates of agreement for each age group (x^2^ = 0.25, *P* = .88) (Fig. 1). Fisher exact test, however, showed a statistically significant 6.4% increase in rates of agreement for female versus male cases (*P* = .003) (Fig. 2).

## Discussion

In evaluating the survey responses of participants, the calculated Cohen kappa supports the study hypothesis that the absence of the oblique view did not significantly change the selected treatment option by the participating physicians over all included cases. Cohen kappa is a test that evaluates the consistency with which participants answer the same question the same way. For the purpose of this analysis, the 2- and 3-view surveys were treated as the same survey taken twice. Any positive Cohen kappa value signifies an agreement between the 2 surveys. As kappa approaches 1, there is a higher degree of agreement. All paired questions in this study had positive kappa values, indicating a positive degree of agreement between the 2 surveys. Twenty-six of these pairings had a kappa score of greater than 0.41, signifying moderate-to-perfect agreement. It should be noted that Cohen kappa is asymptotic, and any question pairing that has a value of “N/A” was in perfect agreement. However, when the paired questions were examined separately, there were many pairs of questions where kappa was lower moderate. Most notably, the 3 cases with a kappa score of less than 0.41 call into question whether it is fully beneficial to omit the oblique view.

The individual question pairings may be limited in their application to this study, based on the review by Bujang and Bharum.^8^ This article describes the guidelines of the minimum sample size required for Cohen kappa when examining crosstabulations larger than 2 × 2. This study worked with a 4 × 4 design, and 32 responses for each individual question may not allow for a sample size large enough to fully support the findings from each individually calculated kappa score. However, if the paired question data set was only examined collectively, the authors would be inclined to support the omission of the oblique view. Based on the suggestions from this article, some of the individually paired questions carry the required sample sizes based on their calculated kappa score; however, many do not.

The above findings suggest that the population of surveyed hand surgeons significantly altered their initial intervention for the management of 3 of the 30 closed DRFs included in the survey. Although participants answered the remaining 27 paired questions similarly, the benefits of ordering the additional view for the entire population likely still outweigh the risks associated with radiation exposure and the minimal financial burden.

The nature of the consecutive surveys used in this study leaves the potential for confirmation bias. Because most participants completed the 2- and 3-view surveys consecutively, subjects potentially recognized the demographic information from the previous survey and chose the answer that agreed with their previous selection. However, the randomization of the order in which cases were presented reduced the potential for such bias. Future renditions of this study should include a mandatory waiting period between surveys to decrease the potential for confirmation bias. The tradeoff here, however, is a likely decrease in complete participation.

In the setting of initial intervention for closed wrist trauma, physicians generally have a low threshold for ordering x-rays to rule out fractures. As previously stated, the 3-view wrist series is the current standard for this purpose. Although preceding studies in the literature have concluded that the oblique view provides additional diagnostic acuity and may reveal fractures not visible on PA and lateral views, the goal of this study was to evaluate the clinical significance of the oblique view in determining treatment modalities in the setting of confirmed DRF.^9^ This survey was designed to isolate the oblique view as the only independent variable between the 2 surveys. To accomplish this, randomly selected cases of DRFs were chosen for inclusion. In doing this, however, the survey created a premise that does not accurately reflect a realistic clinical scenario. When ordering the initial radiographs, the provider does not know the diagnosis. A future rendition of the survey will include radiographs of patients that present with closed traumatic wrist pain regardless of their ultimate fracture diagnosis.

It should again be stated that this study only comments on the clinical value of the oblique view in the specific setting of confirmed, isolated, DRFs. This study in no way assesses the value of the oblique view in the setting of compound fractures or multiple fractures involving the hand, wrist, or forearm. Additionally, the study does not comment on any alternate views in the initial assessment of wrist trauma. For example, many hand specialists order an additional PA view in which the angle of the beam parallels the volar tilt of the radius (normally 10°–25°) to visualize the intra-articular involvement. The nature of this study intentionally did not represent a realistic clinical scenario. When a patient presents with a traumatic wrist injury, one cannot certainly diagnose an isolated distal radius fracture with history and physical alone; therefore, one cannot order films on the basis of a known diagnosis. Instead, this survey is a foundational study designed to evaluate whether the oblique view was clinically valuable in controlled patient and injury groups. With the knowledge gained, the next step is to revisit data to better understand the nuances of the cases that benefited from the inclusion of the third view.
